# Ocular complications of antineoplastic therapies

**DOI:** 10.2144/fsoa-2022-0081

**Published:** 2023-06-01

**Authors:** Abbas Bader, Madeline Begemann, Ammar Al-Obaidi, Muhammad Hamza Habib, Faiz Anwer, Shahzad Raza

**Affiliations:** 1 University of Missouri Kansas City School of Medicine, 5000 Holmes St, Kansas City, MO 64110, USA; 2 Saint Luke's Hospital of Kansas City, 4401 Wornall Rd, Kansas City, MO 64111, USA; 3 Rutgers Cancer Institute of New Jersey, 195 Little Albany St, New Brunswick, NJ 08901, USA; 4 Cleveland Clinic Taussig Cancer Institute, 9500 Euclid Avenue, Cleveland, OH 44195, USA

**Keywords:** antineoplastic agents, cancer, chemotherapy, ophthalmic side effects

## Abstract

Ocular complications of antineoplastic agents can have a profound effect on the quality of life of cancer patients. New oncologic treatments like monoclonal antibodies, immunotherapies, antibody-drug conjugates, checkpoint inhibitors and growth factor receptors have resulted in increased ocular complications. These ocular complications differs in respect to distinct mechanisms of actions and lead to significant challenges in the management of cancer patients. In this review, we reviewed literature, clinical studies and cases detailing ocular complications due to administration of antineoplastic agents and emphasized the need for communication between oncologists and ophthalmologists toward early detection and management of ocular complications.

## Plain language summary

Ocular side effects can have a large impact on the quality of life on patients with cancer. New ways to treat cancer greatly increased patient prognosis, however, it has also resulted in increased ocular complications. This paper aims to review past medical literature, clinical studies, and case reports to describe the types of ocular complications that may arise from administration of antineoplastic agents. This paper emphasizes the need for communication between oncologists and ophthalmologists toward early detection and management for better patient care.

Antineoplastic therapies have been documented to produce a myriad of side effects. Ocular side effects to antineoplastic therapies are often perceived as extremely rare and benign, however, past medical literature seems to suggest that they are relatively more common and more severe. Many of these complications can have long-lasting effects on the patient and are not easily preventable or alleviated. It is recommended that ophthalmologists perform a baseline examination and common periodic follow-ups with patients undergoing therapy in order to recognize and treat adverse ocular side effects that may arise from antineoplastic agents. This review paper aims to give a holistic review of different antineoplastic agents and their adverse ocular side effects. PubMed, Google Scholar, and Science Direct were used to research literature surrounding antineoplastic therapies and potential ocular side effects. We did not use an automated technique and instead manually searched pertinent keywords. These keywords included “ocular toxicity”, “ocular complications”, as well the names of the antineoplastic therapies covered in the paper. This paper used Medical Subject Heading (MeSH) method with a Boolean technique to analyze the keywords. The review paper followed the Preferred Reporting Items for Systematic Reviews and Meta-Analyses (PRISMA) guidelines 2020. While the list of antineoplastic agents is not exhaustive, this review paper aims to include agents that are commonly used in practice. These agents will be organized under the different mechanisms they employ to achieve their antineoplastic effect.

## Chemotherapy

### Alkylating agents

As one of the most common antineoplastic drugs, alkylating agents act upon cancer cell DNA and thereby exploit the decreased activity of DNA repair within cancer cells [1]. Alkylating agents suppress tumor growth by abnormal base pairing, DNA strand breaks, and stimulating DNA cross linking. This largely inhibits cell replication in cancer cells as a result from their high replication rate [1].

#### Cisplatin

Cisplatin is a first-line chemotherapeutic agent that functions through creating intra-strand DNA adducts thus disrupting cell replication and DNA synthesis. Through cisplatin-induced DNA damage, other signal transduction pathways are induced helping suppress tumor growth [2].

Ocular side effects of cisplatin appear to be correlated with cisplatin-related neurotoxicity in relation to dosage amounts above 600 mg/m^2^ [3]. A wide variety of ocular side effects have been documented in past literature. In a study with 13 patients diagnosed of ovarian carcinoma by Wilding *et al.*, 62% of patients developed blurred vision with 23% of patients having altered color perception [4]. Bilateral retrobulbar neuritis and optic neuritis have been reported from high dosage amounts of cisplatin [5]. High dosage intravenous regimens have also been documented to cause macular pigmentary changes, transient cortical blindness [6], and temporary homonymous hemianopia [7]. Cisplatin is also known to cause photopsia, ocular pain, and cystoid macular edema [3]. Ocular toxicity from cisplatin, while rare, is shown to be nearly irreversible. The pathophysiology behind the ocular toxicity of cisplatin is not well elucidated but is hypothesized to involve ischemic and electrophysiological changes within the retina or optic nerve [3]. There is no specialized protocol to treat ocular toxicity secondary to cisplatin, however, flunarizine was reported to successfully treat one patient with visual abnormalities [3]. Clinicians should be aware of these toxicities particularly loss of color vision.

#### Carboplatin

Carboplatin functions in a similar way to cisplatin as both agents are platinum analogs however the mechanism of action of carboplatin is not fully understood. Carboplatin is excreted at a lower rate in the body giving this agent a higher retention half-life [8].

Although rare, symptoms of bilateral metamorphopsia, reduced visual acuities, bilateral disc swelling, and macular oedema have been documented from carboplatin treatment [9]. Abnormal ocular motility has also been noted with subtenon injection of carboplatin [10]. Bilateral ischemic retinopathy was also seen in a combination therapy of carboplatin and paclitaxel. Treatments regarding severe ischemic retinopathy are limited to pausing treatment or pan retinal laser treatment [11]. Ying *et al.* described that corticosteroids and topical anti-inflammatory medications may be helpful for treatment of the side effects [3].

#### Cyclophosphamide

This agent is a nitrogen mustard derivative of alkylating agents. The agent's antineoplastic effects are a result from its conversion to hydroxycyclophosphamide and then to aldophosphamide from hepatic enzymes including cytochrome P-450. This metabolite is then cleaved to form phosphoramide mustard facilitating cross links between guanines in cell DNA [12].

Ocular side effects observed from cyclophosphamide include blurred vision, epiphora, blepharoconjunctivitis, keratoconjunctivitis sicca, and pinpoint hemorrhages [13]. Episodes of reversible blurred vision have been documented to occur within 24 hours intravenous injection at doses of 750 mg/m^2^ with an incidence rate of 17% [14]. Likewise, a case of irreversible lacrimal duct stenosis was documented [15]. Antihistamine tears may help alleviate some of the symptoms of conjunctivitis.

#### Carmustine

Carmustine is a nitrosourea derivative and thus has a unique ability to cross the blood–brain barrier due to their lipophilic nature. Therefore, carmustine is used considerably to treat malignant brain tumors. Carmustine is a nonspecific alkylating agent and carbamates glutathione reductase.

There have been many ocular side effects associated with carmustine reported in past literature. After reviewing 50 records of patients who underwent a high dose carmustine regimen, Shingleton *et al.* found nine cases (18%) of ocular toxicity including symptoms of bilateral nerve fiber-layer infarcts, bilateral field cuts, cloudy and decreased vision, retinal and disc hemorrhaging [16]. It appears that ocular toxicity from carmustine is secondary to segmental intraretinal vasculitis [16]. Symptoms of retrobulbar pain, loss of depth perception, diplopia, optic neuritis, and optic atrophy have also been observed [17,18]. One study found retinal hemorrhages in 24% of patients from a sample size of 110 patients [19]. Chrousos *et al.* attempted infusion of carmustine via intracarotid catheter placed past the beginning of the ophthalmic artery as a means to decrease retinal toxicity. Six patients were treated via this method and none of them developed ocular side effects. While more statistical data needs to be collected to reach a definitive result, it appears the chances of ocular toxicity can be reduced with this procedure [20].

### Antimetabolites

Antimetabolites are compounds that disrupt metabolic processes. This is done primarily through two unique mechanisms of action. They can act as chemical analogs in which they are incorrectly identified and substituted as a different compound in a metabolic pathway thereby disrupting metabolic processes. Most commonly, antimetabolite agents act as analogs and are incorporated within DNA or RNA thus disrupting synthesis. Categories of analogs are separated into base analogs, nucleoside analogs, nucleotide analogs, and antifolates. Antimetabolites may also compete with active site of enzymes via competitive inhibition [21]. Due to the fact that most antimetabolites disrupt DNA synthesis, antimetabolites are effective against rapidly replicating tumor cells as these cells are more often in S-phase.

#### Cytosine arabinoside

Cytosine arabinoside is a pyrimidine analog. Within the cell, this agent competes with cytidine and substitutes itself within DNA hindering replication of DNA. Likewise, cytosine arabinoside hinders DNA polymerase again disrupting DNA synthesis as well as repair.

Ocular side effects to cytosine arabinoside include photophobia, burning ocular pain, blurred vision, and epiphora. Ritch *et al.* documented a case in which a patient undergoing 3.0 g/m^2^ every 12 hours of cytosine arabinoside developed conjunctival injection and central punctate corneal opacities [22]. Two cases of complete visual loss due to optic neuropathy after an intrathecal injection of cytosine arabinoside [23]. Higa *et al.* found that use of prophylactic glucocorticoid eye drops was effective in reducing the incidence chemical conjunctivitis and keratitis. This is proposed to be caused from the dilution of intraocular concentrations of cytosine arabinoside. After high-dose therapy, concentration of cytosine arabinoside was found to be high in CSF, tears, and aqueous humor which may play a role in toxicity [24]. Özcan *et al.* found that administering topical loteprednol etabonate 0.5% helped alleviate toxic keratopathy that arises from high dose cyctosine arabinoside treatment [25].

#### 5-Fluorouracil

5-Fluorouracil is a pyrimidine analog and heterocyclic aromatic compound. The structure of 5-fluorouracil is very similar to uracil allowing for the agent to be incorporated within DNA or RNA promoting cytotoxic effect within cells. Like other antimetabolites, 5-Fluorouracil is especially effective in replicating cells [26].

Different ocular side effects have been reported with 5-fluorouracil. These symptoms range from blurred vision, loss of vision, ocular pain, photophobia, epiphora, eye irritation, conjunctivitis, circumferential edema, ectropion, and keratitis [27,28]. In a study of 18 patients that underwent a high dose regimen of 5-Fluorouracil, permanent intractable epiphora was found as a result of a development of canicular fibrosis [29]. In a case study of 52 patients, canicular stenosis was found in 5.8% of patients and epiphora in 26.9% of patients [30]. Bukhari *et al.* describes a case where a patient was treated corneal epithelial hyperplasia with cyclosporine 1% [31]. Raina *et al.* was able to effectively treat loss of vision on the right eye via intravenous administration of 1 g of methylprednisolone for five days [32]. Christophidus *et al.* observed that ocular effects occurred when a detectable concentration of 5-flourouracil was found in the lacrimal fluid of affected patients [33]. Within most of the literature, cessation of 5-fluorouracil prevented further exacerbation of symptoms and helped alleviate them over time.

#### Other drugs

Several other alkylating agents, antimetabolites, antimicrotubules, and anthracyclines can cause distinct ocular toxicities which are described in Table 1 [34–45].

**Table 1.** Chemotherapy.

Chemotherapy	Drug name	Symptoms	Ref.
Alkylating agent	Mechlorethamine	Necrotizing uveitis	[34]
	Chlorambucil	Diplopia, retinal hemorrhages, papilledema	[35]
	Ifosfamide	Blurred vision, florid conjunctivitis	[36]
	Oxaliplatin	Conjunctivitis, blurred vision, ocular pain	[37]
Antimetabolites	Capecitabine	Ocular irritation, blurred vision, corneal deposits	[38]
	Methotrexate	Blepharitis, conjunctivitis, photophobia, periorbital edema, blurred vision	[35]
	Pemetrexed	Visual field defects, ischemic optic neuropathy	[39]
	Fludarabine	Photophobia, diplopia, cortical blindness	[40]
	Gemcitabine	Blurred vision, uveal effusion	[41]
	Pentostatin	Conjunctivitis, keratoconjunctivitis, photophobia, retinopathy	[42]
	Mitomycin-C	Blurred vision	[43]
Microtubules inhibitor	Paclitaxel	Visual impairment, scintillating scotoma	[44]
	Docetaxel	Conjunctivitis	[45]
Anthracyclines	Doxorubicin	Conjunctivitis, epiphora	[43]

## Targeted therapies

### Tyrosine kinase inhibitors

Tyrosine kinases play a significant role as catalytic mediators of multiple cell signal pathways. With more than 90 different tyrosine kinases identified within the human body, these enzymes phosphorylate tyrosine residue groups on certain target proteins via ATP. Tyrosine kinases play key roles within the cell pathways regarding metabolism, differentiation, proliferation, and apoptosis [46]. Abnormal expression and deregulation of tyrosine kinases has been linked with oncogenic signaling which can result from mutations within the genes that code for the protein. Tyrosine kinase inhibitors (TKI) work as competitive inhibitors for the ATP binding site which in turn decreases unregulated cell proliferation [47]. TKIs have been implicated to cause a class effect regarding ocular toxicity including central serous retinopathy, retinal pigment epithelium detachment, and keratitis [48].

#### BCR-ABL inhibitors

The BCR-ABL gene is a fusion between a tyrosine kinase gene and breakpoint cluster gene causing constitutive tyrosine kinase activity. The BCR-ABL gene is caused by a reciprocal translocation [t(9;22)(q34;q11) and is commonly known as the Philadelphia chromosome. This fusion is a hallmark of chronic myeloid leukemia but can also be seen in acute lymphoblastic leukemia and chronic neutrophilic leukemia [49].

##### Imatinib

Imatinib is a 2-phenyl amino pyrimidine derivative that acts as a competitive inhibitor of multiple tyrosine kinase isozymes by binding to the ATP active site. Imatinib is especially effective against reducing a constitutively active tyrosine kinase activity caused by a BCR-ABL merged gene caused by the Philadelphia chromosome. Imatinib is also used to target the genes proto-oncogene c-KIT (KIT) and platelet-derived growth factor receptors (PDGF-R) that also cause abnormal tyrosine kinase activity [50].

According to the FDA and FDA-cited sources, multiple ocular side effects can be observed in administration of imatinib. Common side effects include periorbital edema, hyper lacrimation, and visual disturbance. Other ocular side effects include conjunctivitis, conjunctival hemorrhage, retinal hemorrhage, increased intraocular pressure, epiphora, blurred vision, and keratoconjunctivitis [51,52]. These results are found in literature as well. According to Fruanfelder *et al.*, in a chart review of 104 patients, 18% developed epiphora and about 70% of patients develop periorbital edema which usually starts 5–8 weeks after initial administration of imatinib [52]. While imatinib induced periorbital edema is usually mild, severe cases of periorbital edema have been treated with blepharoplasty [53]. Analysis of previous literature suggests that incidence of periorbital edema is correlated with increasing dosage amounts. In a prospective study of 54 patients who were administered with imatinib, it was found that the average dosages of patients with periorbital edema (471 ± 113 mg) were higher than patients who did not suffer these symptoms (395 ± 108 mg) [52]. One case reported vitreous hemorrhage with a dosage of 400 mg/day. A possible mechanism of toxicity could involve inhibition of a significant portion of c-KIT positive mast cells present within conjunctival mucosa [54]. Oral diuretics and topical steroid eye drops may help treat periorbital edema and epiphora [52].

#### Fibroblast growth factor receptors inhibitors

##### Pemigatinib

Pemigatinib is a fibroblast growth factor receptor (FGFR) selective inhibitor for isozymes 1–3. This is a tyrosine kinase that is involved in activating metabolic pathways that help cell proliferation, growth, and differentiation. As a result, pemigatinib is commonly used for cancers that upregulate FGFR expression [55]. Pemigatinib is frequently administered for patients undergoing treatment for cholangiocarcinoma and colorectal cancer.

Medical literature detailing ocular toxicity from pemigatinib administration is mostly related to serous retinopathy and subretinal fluid accumulation. In a case reported by Alekseev *et al.*, a 67-year-old male being treated with pemigatinib for a stage IVA colorectal cancer developed bilateral multifocal serous retinopathy after 42 days of oral administration (13.5 mg daily) of the drug [56]. Pemigatinib was immediately discontinued and symptoms resolved within 5 days. Bloom *et al.* also details a case of subretinal accumulation and subsequent serous retinal detachment from treatment of pemigatinib. Interestingly, symptoms of blurred vision and increased floaters second to subretinal accumulation manifested within 8 days of treatment. Treatment was withheld and symptoms resolved within 2 weeks. However, after resuming administration of pemigatinib at a lower dose of 9 mg a day, symptoms resurfaced again [57]. More severe complications can also surface such as macular edema, retinal hemorrhage, and retinal vein occlusion. In the PACE trial, retinal toxicity occurred in 3.6% of patients [58]. Analysis of literature regarding other FGFR inhibitors dictates that FGFR inhibitors may broadly have a class effect on serous retinopathy [59]. Alekseev *et al.* found abnormal electrooculogram findings as well as anti-retinal pigment epithelium antibodies and hypothesized that a possible mechanism of toxicity could involve retinal pigment epithelium pump dysfunction [56]. There is no specific protocol for treatment for pemigatinib induced ocular side effects and individualized treatments should be developed by the ophthalmologist. However, cessation of pemigatinib has shown to alleviate ocular toxicity.

#### Bruton tyrosine kinase inhibitors

Bruton tyrosine kinase (BTK) are a cytoplasmic receptor that helps facilitate transduction of other cell surface receptors. Most commonly, BTKs are found in hematopoietic cells and have five signaling domains. This allows for a wide variety of partner molecules to bind to the BTK. Most BTKs are highly expressed within B cells, myeloid cells, and platelets [60]. This allows them to function in antigen receptor signaling. As a result, mutations in BTK encoding genes result in a variety of tumors emphasizing the importance of BTK inhibitors as an antineoplastic agent.

##### Ibrutinib

Ibrutinib is an irreversible bruton tyrosine kinase inhibitor that is commonly used in diffuse large B-cell lymphoma, follicular lymphoma, multiple myeloma, and pancreatic cancer. Ibrutinib, when ingested orally, will covalently bond to cysteine residues within the active site of bruton kinase inhibitors at cysteine residue-481. Ibrutinib also prevents autophosphorylation of at tyrosine residue 223 inhibiting the downstream B cell antigen pathway thus impeding proliferation of potential B-cell tumor cells.

Instances of ibrutinib ocular side effects are documented in medical literature. A case of severe sclerouveitis was described in a case study by Mehraban *et al.* [61]. This is likewise corroborated by Bohn *et al.* where they reported two severe cases of ibrutinib-related uveitis [62]. One patient was reported with an acute onset bilateral fibrinous anterior uveitis along with persistent subretinal fluid and hyperemic discs. Unlike the previous case study by Far *et al.*, ocular related symptoms were reported a day after administration of ibrutinib. The other patient presented with bilateral hypertensive anterior uveitis with right eye hyphemia and pupillary seclusion during his 9 months of ibrutinib administration. In both cases, ocular side effects faded after administration of ibrutinib was halted [54,61]. A correlation between ibrutinib and cataracts has also been explored within literature. In a study by Bryd *et al.* with a sample size of 391, Ibrutinib was found to have a cataract incidence rate of 3% [63]. Administration of oral and local topical steroids can be effective in treating symptoms, however, Bohn *et al.* describes a case in which a patient reacted poorly to steroids and cessation of ibrutinib was ordered [62]. Fu *et al.*, recommends screening with a slit lamp and dilated fundoscopic exam should be conducted [64].

#### BRAF inhibitors

BRAF inhibitors are a targeted therapy that act upon BRAF kinase. The BRAF kinase plays an important part in conveying a downstream signal toward the mitogen activated protein kinase (MAPK) pathway. The MAPK pathway is known to regulate cell growth and proliferation. BRAF inhibitors are commonly used in melanomas where the BRAF kinase may become mutated to become overactive [65].

##### Vemurafenib

Vemurafenib is a specialized inhibitor that blocks a specific mutation of BRAF kinase. Vemurafenib does not inhibit wild-type BRAF kinase and instead inhibits mutated BRAF V600E that is found in several types of cancer. Like other BRAF inhibitors, vemurafenib inhibits overactivation of the MAPK pathway and thus inhibits tumor proliferation, cell growth, and angiogenesis [65].

In a retrospective review conducted by Choe *et al.*, which analyzed 568 patients that were administered with vemurafenib, 22% of the patients developed ocular side effects. Common side effects that developed from administration of vemurafenib include uveitis (4%), blurred vision (3.7%), ocular discomfort (3.5%), photophobia (3%), conjunctivitis (2.8%), and keratoconjunctivitis (2.1%). Although rare, some patients developed more severe complications such as unilateral ischemic central retinal vein occlusion (0.2%), double vision (0.9%), and persistent ocular hyperemia (0.2%). Uveitis was able to be effectively managed with use of corticosteroids, cycloplegic agents, along with agents that lower intraocular pressure without ceasing or lowering dosage of vemurafenib [66]. Vemurafenib is capable of crossing the blood–brain barrier and also the blood retinal barrier. A possible mechanism of vemurafenib induced uveitis could involve vemurafenib activity on subclinical metastatic cells inducing lymphocytic infiltration and inflammation [66].

#### Mitogen-activated protein kinase Inhibitors

Mitogen-activated protein kinase (MEK) inhibitors are a specific targeted therapy that disrupt and inhibit the normal MAPK pathway. As mentioned before, the MAPK pathway has a strong influence on cell differentiation, proliferation, and apoptosis [67]. MEK inhibitors are often used along with BRAF inhibitors to treat a variety of cancers including melanomas.

##### Trametinib

Tremetinib was approved in 2013 to treat metastatic melanoma with BRAF V600E/K mutations [68]. Trametinib is an allosteric inhibitor of MEK1/MEK2 activation and kinase ability which could lead to G1 cell cycle arrest and cell apoptosis.

In a phase I clinical trial consisting of 206 patients, 15% of patients developed ocular side effects. About 1.5% percent of patients in the study developed central serous retinopathy and one patient developed retinal vein occlusion. In phase III clinical trials, about 9% of the patients developed ocular side effects. The most common symptom was blurred vision [69]. Medical literature suggests that a class effect of MEK inhibition may include retinal vien occlusion and serious retinopathy [70]. Duncan *et al.* hypothesizes that MEK inhibition may influence the permeability of the retinal pigment epithelium and may cause subretinal fluid accumulation [69]. In a case study by Sarny *et al.*, uveitis and neuro retinal detachment was found secondary to combination therapy of trametinib and dabrafenib and was treated with topical corticosteroids after cessation of trametinib and dabrafenib [71].

#### Anaplastic lymphoma kinase inhibitors

Anaplastic lymphoma kinase inhibitors are used to inhibit the anaplastic lymphoma kinase (ALK). Fusions of ALK proteins can lead to downstream overactivation of the JAK/STAT pathway as well as the MAPK pathway leading to cell proliferation, differentiation, and spread [72].

##### Crizotinib

Crizotinib is a concentration dependent inhibitor of ALK prevents which prevents tumour cell proliferation and differentiation.

In phase II clinical trials in which patients were administered 250 mg, nearly 65% of patients were documented to have visual disturbances such as photophobia, blurred vision, flashes, and trailing lights. Compared with other ALK inhibitors, crizotinib is noted to have a higher incidence rate of ocular side effects [73]. Crizotinib has also been reported to cause eyelid abnormalities, retinal vein occlusion, retinopathy [74]. There is no specialized protocol to treat ocular toxicity secondary to crizotinib and treatment should be individualized and done by the ophthalmologist.

### Monoclonal antibodies

Monoclonal antibodies are proteins designed to recognize and bind to specific antigens expressed on a target cell. Once bound to the target cancer cell, monoclonal antibodies have a variety of ways of reducing growth or inducing apoptosis. Some monoclonal antibodies are designed to bind to growth factor receptors preventing division of the tumor cell. Monoclonal antibodies also can carry cytotoxic or radiative molecules to the target cell. These are called conjugated monoclonal antibodies. Natural killer cells can also be induced causing antibody dependent cell mediated cytotoxicity.

#### Pembrolizumab

Pembrolizumab is an immune checkpoint inhibitor which aims to increase the effectiveness of T-cells by inhibiting immune checkpoint proteins that inhibit T-cell function. Pembrolizumab is an IgG4 kappa monoclonal antibody that is designed to specifically target PD-1 and block its complex with PDL-1 on T-cells leading to increased performance. It is primarily used in melanoma and non-small cell lung cancer but is approved to be used in a variety of other cancers [75].

Ocular side effects to pembrolizumab have been documented in past medical literature to include conjunctivitis, uveitis, keratitis, orbitopathy, retinal vasculitis, and choroiditis [76]. keratoconjunctivitis have been documented as the most common side effect. Nguyen *et al.* describes a case study regarding a 57-year-old patient who developed ocular hypotony and near complete vision loss secondary to pembrolizumab administration [77]. Adverse ocular side effects to pembrolizumab usually arise weeks to months after initial administration. Side effects may be treated with topical or systemic corticosteroids, artificial tears, or topical cyclosporine [76].

#### Panitumumab

Panitumumab is a competitive inhibitor of the extracellular binding domain of EGFR. This leads to decreased binding of ligands thus reducing receptor autophosphorylation and subsequent downstream signaling of protein kinases. Some cancers cause the EGFR to become overactive and cause uncontrolled proliferation of tumor cells as neo-angiogenesis. Panitumumab can decrease tumor growth, angiogenesis, and spread [78,79]. Panitumumab is commonly used to treat colorectal cancers.

Ocular side effects are fairly well described within medical literature detailing panitumumab and other EGFR inhibitors. It is believed that ocular toxicity secondary to this class of drugs are due to insufficient EGFR signaling to different ocular tissues [80]. In the phase III trial of panitumumab, multiple ocular side effects were noticed including conjunctivitis, conjunctival hyperemia, increased lacrimation, and irritation [81]. There is no specialized protocol to treat ocular toxicity secondary to panitumumab and treatment should be individualized and done by the ophthalmologist.

#### Balantamab

Belantamab mafodotin is a conjugated monoclonal antibody used to treat multiple myeloma. Belantamab is a targeted therapy for B cell maturation antigen (BCMA) on plasma cells. More specifically, belantamab gains its specificity to bind to BCMA by its anti-CD38 monoclonal antibody part and its cytotoxicity from maleimidocaproyl monomethyl auristatin F. The specificity, selectivity, and binding affinity balantamab exhibits from being a monoclonal antibody makes it a favorable option to treat multiple myeloma [82].

Ocular side effects of belantamab have been reported within its first in-human trial DREAMM-1. In the first part of the study, there was a 53% incidence rate of keratopathy. This percentage spiked to a 63% incidence rate in the second part of the study. Wahab, Rafae *et al.* propose that the ocular toxicity of the drug arises from the maleimidocaproyl monomethyl auristatin F which has the cytotoxic effect of destabilizing microtubules causing cell cycle arrest within G2/M phase [83]. This has been observed to cause unspecific damage to corneal epithelium resulting in keratopathy. Even if ocular symptoms are not observed, it is still recommended to conduct routine slit lamp tests and visual acuity tests to gauge levels of ocular toxicity. Topical steroids were shown to be ineffective and are not recommended to be used, however, preservative-free artificial tear drops are recommended in alleviating symptoms. Holding belantamab administration until symptoms subside or lessen appears to be the most effective treatment of side effects [83].

#### Rituximab

Rituximab is a monoclonal antibody used to commonly treat non-hodgkin's lymphoma and lymphocytic leukemia [84]. Rituximab particularly targets B-lymphocytes by using and attaching CD20 antigens. Rituximab can induce antibody dependent cell mediated cytotoxicity and complement mediated cytotoxicity. Moreover, while rituximab can cause apoptosis of target tumour cells, it can also make cells more sensitive to other chemotherapeutic drugs [85].

Ocular side effects of rituximab include acute retinal sclerosis, progressive outer retinal necrosis, bilateral conjunctivitis, and macular oedema [86]. Foran *et al.* reports that out of 222 patients taking rituximab, nine of them developed ocular side effects. These included burning sensations in the eye, partial loss of vision, transient ocular edema, and conjunctivitis [87]. There is no specialized protocol to treat ocular toxicity secondary to Rituximab and treatment should be individualized and done by the ophthalmologist.

### Chimeric antigen receptor (CAR) T-cell therapy

Chimeric antigen receptor (CAR) T-cell therapy is a recent therapy that focuses upon modifying T-cells with chimeric antigen receptors in order to alter the surface antigen. The binding domain of the chimeric antigen receptor is usually derived from a single chain variable fragment of an antibody. This gives CAR a wide flexibility to recognize and target other surface antigens including proteins, carbohydrates, and glycolipids. This therapy is commonly used for B-cell acute lymphoblastic leukemia and non-Hodgkin's lymphoma [88].

#### Tisagenlecleucel

Tisagenlecleucel is autologous T-cell immunotherapy that has been modified to express anti-CD20 antigens. Tisagenlecleucel is often used to treat refractory or relapsed B-cell acute lymphoblastic leukemia.

Ocular side effects of tisagenlecleucel have been reported from a retrospective study analyzing data from the FDA's adverse event reporting system. The retrospective study analyzed 1421 patients and 17 of those cases reported ocular side effects. The most common side effects included vision acuity changes in 35.3% of affected patients. Other symptoms included impaired pupil response, papilledema, mydriasis, and photophobia [89,90]. There is no specialized protocol to treat ocular toxicity secondary to tisagenlecleucel and treatment should be individualized and done by the ophthalmologist.

### Proteasome inhibitors

Proteasome inhibitors are a class of therapies that classically manage patients with multiple myeloma and mantle cell lymphoma. Proteasomes play an integral part of in cell survival, DNA repair, and cell proliferation. This is done through the degradation of cyclin dependent kinase which allows the cell to undergo the full cell cycle. Inhibition of this would help against unregulated cell proliferation [91].

#### Bortezomib

Bortezomib is a proteasome inhibitor commonly used to treat patients with multiple myeloma and mantle cell lymphoma. It reversibly binds to chymotrypsin-like 26S subunit of the proteasome and causes inhibition leading to apoptosis via caspase mediated pathways [92].

Ocular toxicity of bortezomib often involves the eyelids and is indicated with chalazia and blepharitis. In a case series of 14 patients, it was approximated that the average time for ocular toxicity to develop from onset of bortezomib administration was around 3 months. Potential treatment options include warm compresses, topical and oral antibiotics as well as lowering dosage or discontinuing medication [93]. Administration of oral doxycycline was found to be helpful in treating Bortezomib-induced blepharitis [94]. Resolution of bortezomib induced ocular toxicity was reported to be around 2 months [93].

#### Other drugs

Further description of ocular complications with other targeted therapy agents are described in Table 2 [60,95–104].

**Table 2.** Targeted therapy agents.

Targeted therapy	Drug name	Signs and symptoms	Ref.
EGFR inhibitor	Cetuximab	Squamous blepharitis, bilateral corneal erosions	[95]
	Afatinib	Unilateral iridocyclitis, bilateral conjunctivitis, anterior uveitis	[96]
	Erlotinib	Blepharitis, conjunctivitis, corneal erosion, uveitis	[97]
	Osimertinib	Corneal epithelial lesions, vortex keratopathy	[60]
FGFR inhibitor	Erdafitinib	Keratoconjunctivitis, blurred vision, cataracts	[98]
BRAF inhibitor	Dabrafenib	Macular edema, unilateral anterior uveitis	[99]
	Encorafenib	Visual impairment, bilateral retinopathy	[100,101]
MEK inhibitor	Binimetinib	Retinal pigment epithelial detachment	[102]
	Selumetinib	Subconjunctival hemorrhage, eyelid edema, diplopia, blurred vision	[103]
	Cobimetinib	Subfoveal neurosensory retinal detachment	[103]
Checkpoint inhibitor	Ipilimumab	Conjunctivitis, scleritis, uveitis	[104]
	Nivolumab	Uveitis	[103]
ALK inhibitor	Lorlatinib	Vision defects	[104]
Tyrosine inhibitor	Dasatinib	Macular edema, optic disc edema, periorbital edema, optic neuritis	[97]

## Conclusion

It is clear to see that administration of antineoplastic agents can result in severe ocular side effects. This review paper placed a particular emphasis upon chemotherapy and targeted therapies, however ocular toxicity is also present within hormonal agents such as tamoxifen which can manifest ocular side effects such as macular edema, corneal opacities, bilateral optic neuritis, retinal hemorrhages [105,106]. Overall, it is critical for oncologists to be cognizant of the potential ocular side effects of treatments and be used in the analysis of burden. Moreover, it is important to realize that these symptoms can arise sub-acutely. Consultation with an ophthalmologist should be done immediately upon finding any ocular symptoms in order to prevent further exacerbation of symptoms. It is recommended that patients undergo a baseline ophthalmic examination before cancer treatment and periodic examinations during treatment in order to detect ocular toxicity. It has also been shown that cessation of the treatment or lowering the dose of the treatment can help reverse severe ocular toxicity. Overall, it is important to have frequent communication between oncologists and ophthalmologists to decrease the risk of developing ocular toxicity by early detection and effectively treating ocular toxicity if it does arise. Similarly, it is important to have open communication between oncologists and patients, especially in very advanced cancers with limited prognosis where such treatments may not add much to the quantity or quality of life, while adding distressing iatrogenic ocular morbidity at the end of life.

## Future perspective

The use of antineoplastic therapies has revolutionized patient care and has greatly reduced cancer mortality. Despite the wide use and effectiveness of these therapies, adverse ocular effects can still be seen. Ocular toxicity has a significant impact on patient quality of life, and it is important to continue to analyze and study cases of ocular complications to antineoplastic therapies. Likewise, it is important to analyze the mechanisms of ocular toxicity. Doing so will allow for better ways to prevent and treat ocular toxicity. Moreover, elucidation of possible mechanisms of ocular toxicities will give insight for future antineoplastic therapies to have less adverse ocular side effects and improve overall patient care.

**Executive summary**

**Background**

- Ocular toxicities have substantial impact on quality of life of the patients.
- With recent advances in cancer treatments which include monoclonal antibodies, immunotherapies, antibody-drug conjugates, checkpoint inhibitors, growth factor receptors, many patients have suffered increase eye toxicities and these toxicities differs in respect to distinct mechanisms of actions.

**Ocular side effects**

- Common symptoms include keratitis, conjunctivitis, and blurred vision.
- We propose baseline examinations and periodic follow-up throughout cancer treatments with known ocular toxicities.
- Many ocular complications to antineoplastic agents can usually be reversed by halting administration or lowering the dosage amount.

**Future perspective**

- It is important to continue to analyze and study cases of ocular complications to antineoplastic therapies in order to better prevent and treat ocular toxicity as well as give insight for future antineoplastic therapies to have less adverse ocular side effects.
